# Pathological amyloid β and tau induce a metabolic switch in cerebral endothelial cells: implications for blood brain barrier dysfunction and perivascular inflammation

**DOI:** 10.1002/alz70855_106671

**Published:** 2025-12-24

**Authors:** Silvia Fossati

**Affiliations:** ^1^ Alzheimer's Center at Temple, Lewis Katz School of Medicine, Philadelphia, PA, USA

## Abstract

**Background:**

Cerebral endothelial cells (cECs) lining the vessel walls regulate blood brain barrier (BBB) and neurovascular unit function, and their failure causes micro‐hemorrhages, neurodegeneration and neuroinflammation processes in Alzheimer's disease (AD). Our lab focuses on understanding the mechanisms by which cECs function is altered by AD and cerebral amyloid angiopathy (CAA) pathology, and how cEC dysfunction mediates BBB and neurovascular alterations. Particularly, we have recently observed that both pathological amyloid β (Aβ) and tau aggregates similarly induce a switch to glycolytic metabolism in cECs and impair endothelial mitochondrial function. Moreover, we have explored how this metabolic switch is linked to endothelial inflammatory activation and BBB permeability.

**Method:**

We analyzed mitochondrial respiration and glycolysis in human cEC challenged with pathological Aβ and tau aggregates, by Seahorse Extracellular Flux Analyzer and related assays. We assessed BBB function as trans‐endothelial electrical resistance (TEER) in real time, using the ECIS Zθ system. Endothelial inflammatory activation and related vascular dysfunction pathways were measured using an MSD V‐Plex Neuroinflammatory Panel, WB and immunofluorescence. Moreover, we explored how glycolysis inhibition rescues BBB and neurovascular dysfunction, and we replicated some of these findings in respective animal models of cerebral amyloidosis or tauopathy.

**Result:**

Both Aβ toxic vasculotropic species and tau protofibrillar aggregates promote a metabolic shift towards glycolysis in cEC, which mediates inflammatory activation and BBB permeability. We also discovered that reduction of glycolysis back to control levels can revert the detrimental cerebrovascular effects of AD pathological aggregates. Interestingly, while Aβ induces a direct switch from mitochondrial respiration to glycolysis in cECs, tau protofibrils hyperactivate glycolysis early, instigating inflammatory EC activation and BBB permeability, while mitochondrial respiration fails after longer tau challenges, closely preceding cEC death. Key data were confirmed in animal models of cerebral amyloidosis or tauopathy.

**Conclusion:**

These studies reveal a new and specific metabolic mechanism that mediates endothelial inflammatory activation and BBB pathology induced by both Aβ and tau toxic species, providing novel possible therapeutic avenues to limit endothelial activation, adhesion/migration of immune cells into the brain and BBB permeability in AD, CAA and tauopathies.

